# Similar perceived availability but lower use of physical activity spaces among Chilean adults with disabilities

**DOI:** 10.3389/fspor.2026.1969080

**Published:** 2026-10-14

**Authors:** Josivaldo De Souza-Lima, Ciro Winckler, Jurgi Olasagasti-Ibargoien, Rodrigo Yáñez-Sepúlveda, Cristian Godoy-Tapia, Maribel Parra-Saldías, Daniel Duclos-Bastías, Andrés Godoy-Cumillaf, Eugenio Merellano-Navarro, José Bruneau-Chávez, Tomás Reyes-Amigo, Claudio Farías-Valenzuela

**Affiliations:** 1 Facultad de Educación y Humanidades, Escuela de Ciencias del Deporte, Universidad Andrés Bello, Santiago, Chile; 2 Programa de Pós-Graduação em Ciências do Movimento Humano e Reabilitação, Universidade Federal de São Paulo (UNIFESP), Santos, São Paulo, Brazil; 3 Health, Physical Activity and Sports Science Laboratory, Department of Physical Activity and Sports, Faculty of Education and Sport, University of Deusto, Bilbao, Spain; 4 Universidad Espíritu Santo, Samborondón, Ecuador; 5 Facultad de Educación, Carrera de Educación Física, Universidad Adventista de Chile, Chillán, Chile; 6 Departamento de Educación Física, Deporte y Recreación, Universidad de Atacama, Copiapó, Chile; 7 iGEO Research, Escuela de Educación Física, Pontificia Universidad Católica de Valparaíso, Valparaíso, Chile; 8 METIS Research Group, Facultad de Negocios y Tecnología, Universidad Alfonso X el Sabio (UAX), Madrid, Spain; 9 Grupo de Investigación en Educación Física, Salud y Calidad de Vida (EFISAL), Facultad de Educación, Universidad Autónoma de Chile, Temuco, Chile; 10 Department of Physical Activity Sciences, Faculty of Education Sciences, Universidad Católica del Maule, Talca, Chile; 11 Departamento de Educación Física, Deportes y Recreación, Universidad de La Frontera, Temuco, Chile; 12 Physical Activity Sciences Observatory (OCAF), Universidad de Playa Ancha, Valparaíso, Chile; 13 Escuela de Ciencias de la Actividad Física, Universidad de Las Américas, Santiago, Chile

**Keywords:** accessibility, built environment, disability, national survey, physical activity

## Abstract

**Background:**

Adults with disabilities are less physically active than the general population, yet the environmental point at which this disparity emerges is rarely identified in national surveillance. We examined perceived availability, use, and barriers to the use of physical activity spaces among Chilean adults with and without disabilities, and estimated the association of disability with physical inactivity and with non-use of available spaces.

**Methods:**

Cross-sectional analysis of the 2024 National Survey of Physical Activity and Sports Habits (ENAFyD), a probabilistic national sample of adults in Chile (*n* = 8,653). Self-reported disability was classified into mutually exclusive categories (sensory only, physical only, cognitive only, multiple types, other only, none) and cross-classified with perceived availability of spaces near the home, their use, reported barriers among non-users, and the survey physical activity index. Group differences were tested with weighted Poisson regression with robust variance, reported as crude prevalence ratios (PR) with 95% confidence intervals and *p* values; adjusted models included sex, age group, and socioeconomic status, selected *a priori* as confounders using a directed acyclic graph.

**Results:**

The weighted prevalence of disability was 9.3%. Perceived availability of spaces did not differ between groups (92.1% vs. 91.4%; crude PR 1.01; 95% CI 0.99-1.03; *p* = 0.478). However, among adults with available spaces, only 29.9% (95% CI 25.4-34.8) of those with disabilities used them, compared with 42.7% (40.8-44.6) of those without (crude PR for non-use 1.22; 1.16-1.29; *p* < 0.001). The largest differential barriers were distance or difficulty of access (14.0% vs. 7.7%; PR 1.82; 1.44-2.31) and lack of companionship (25.9% vs. 16.8%; PR 1.54; 1.31-1.80). In adjusted models, disability was associated with physical inactivity (PR 1.22; 1.11-1.33) and with non-use of available spaces (PR 1.14; 1.08-1.20), with the largest estimate among adults reporting multiple disability types (PR 1.47; 1.35-1.60).

**Conclusions:**

In Chilean adults, the disability gap in physical activity is concentrated in the use of spaces rather than in their perceived availability, and access- and companionship-related barriers were more frequently reported by non-users with disabilities. Policies should prioritize the usability of existing spaces, through accessibility, safe access, and social support, alongside new construction.

## 1 Introduction

Physical inactivity remains one of the leading modifiable risk factors for noncommunicable diseases and premature mortality worldwide, with approximately one third of adults failing to meet the World Health Organization recommendation of at least 150 min of moderate to vigorous physical activity per week (1, 2). For people living with disabilities, the stakes are considerably higher. An estimated 1.5 billion people worldwide live with a physical, mental, sensory, or intellectual disability, and they are 16% to 62% less likely to meet physical activity guidelines than people without disabilities, while facing a higher risk of inactivity-related health problems (3). Recognizing this, the World Health Organization released in 2020 the first global physical activity guidelines applicable to people living with disability, emphasizing that the health benefits of physical activity generally outweigh the risks and calling for inclusive policies and environments (4), in line with the right to full participation enshrined in the United Nations Convention on the Rights of Persons with Disabilities (5).

The built environment is a central determinant in this equation. Access to parks, plazas, cycle paths, and sports facilities near the home is consistently associated with higher physical activity in general populations, and a systematic review concluded that built environment attributes moderate the relationship between having a disability and lower levels of physical activity (6). However, for people with disabilities the mere existence of such spaces may not translate into use: audits of fitness and recreation facilities continue to document substantial accessibility deficits (7), barriers related to distance, safety, and the need for companionship or assistance can convert nominally available infrastructure into functionally inaccessible environments (8), and a synthesis of 22 review articles identified more than 200 barriers and facilitators operating from the individual to the policy level (9).

Framing matters for prevention policy: physical activity promotion for people with disabilities belongs to the population-wide prevention agenda rather than to a rehabilitation niche. The World Health Organization applies its physical activity guidelines to people living with disability (2, 4), and Chile's new National Policy on Physical Activity and Sport 2026-2037 explicitly acknowledges, citing the national disability survey, that 79.3% of persons with disabilities engage in no physical activity (10). National surveillance systems should therefore be able to monitor this group within general population instruments, which is what the present study explores.

In Latin America, evidence on physical activity among adults with disabilities is limited (11). In Chile, the only national analysis available, conducted during the COVID-19 pandemic in a disability-specific survey, found that just 11.9% of adults with disabilities met physical activity guidelines and 62.6% reported no physical activity at all (11), while socioeconomic inequalities in physical activity have been documented for the general population (12) and measurement design itself shapes who appears inactive in national data (13). However, national surveillance systems rarely allow environmental access indicators such as availability, use, and barriers to be disaggregated by disability status. The 2024 National Survey of Physical Activity and Sports Habits (ENAFyD 2024) offers this opportunity: it collected, in a probabilistic national sample of adults, self-reported disability across sensory, physical, and cognitive domains, together with perceived availability of spaces for physical activity near the home, their actual use, and the reasons for non-use (14). Our team has previously used this survey to examine correlates of inactivity in adults (15) and movement behaviors in children and adolescents (16).

Therefore, the aim of the present study was to examine perceived availability, use, and barriers to the use of physical activity spaces among Chilean adults with and without disabilities, and to estimate the association of disability with physical inactivity and with non-use of available spaces.

## 2 Materials and methods

### 2.1 Study design and data source

This is a cross-sectional analysis of the ENAFyD 2024, a national household survey conducted by the Chilean Ministry of Sport (14). The survey used a probabilistic, stratified sampling design covering urban and rural areas of the 16 regions of the country, with strata defined by age band (5-17 and 18 years and over), sex, geographic zone (urban, rural), and socioeconomic status. The sampling frame was the 2017 national census (16,407,857 people aged 5 years and over). Sample size was allocated equally across regions (785 cases per region, and 1,028 in the Metropolitan Region), yielding margins of error of 3.5% by region, 3.06% for the Metropolitan Region and 0.87% nationally; people living in areas defined as difficult to access by the National Institute of Statistics were excluded from the target population. Because the regional allocation is deliberately disproportionate to population size, survey weights are required for national estimates, and this design feature underlies the design effect reported in Section 2.4. Trained interviewers conducted face-to-face fieldwork between October and December 2024 using mobile devices. The total survey comprised 12,803 cases; the present study uses the adult database (*n* = 8,653). We applied a normalized survey weight to all estimates.

### 2.2 Participants and ethics

All adult respondents were included (*n* = 8,653). The adult sample comprises people aged 18 years and over and therefore includes older adults, who represent a substantial share of the group with disabilities (Section 3.1). The flow of participants through each analysis is shown in Figure 1. The ENAFyD 2024 was reviewed and approved by the Ethics Committee constituted for the survey, in accordance with the Declaration of Helsinki; the identifier 799595-2-LQ24 corresponds to the public tender of the Subsecretaría del Deporte (Undersecretariat of Sports), Chilean Ministry of Sport, under which the survey was commissioned. Adult participants provided verbal informed consent following a protocol approved by the committee, and the committee resolution explicitly authorizes the use of the data for academic studies and articles. The present study used anonymized secondary data and required no additional approval.

**Figure 1:**
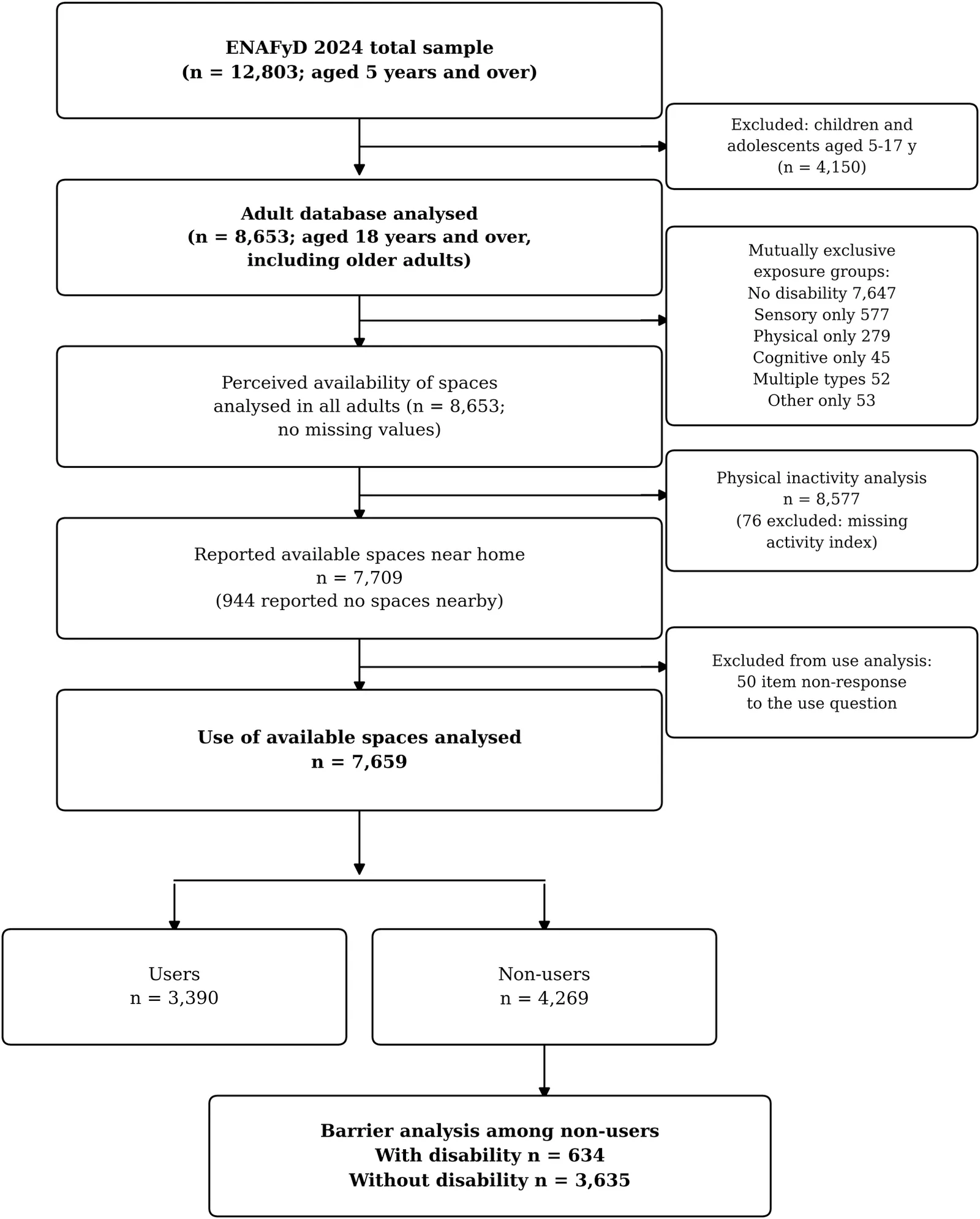
Flow of participants through the analyses, ENAFyD 2024.

### 2.3 Variables

All variables were obtained from the structured questionnaire administered by trained interviewers as part of the ENAFyD 2024; the present study analysed selected variables from the anonymized public database, and the instrument is documented in the survey executive report (14). Following an epidemiological framework, we specify below the independent variable (exposure), the dependent variables (outcomes), and the covariates, with their analytical categories. The assumed relationships among them are summarized in Supplementary Figure 1.

#### 2.3.1 Independent variable (exposure): disability

Participants self-reported whether they had any of the following disabilities (yes/no for each): visual, hearing, physical, cognitive/intellectual (hereafter cognitive), other, or none; reports were not verified against clinical or administrative records, and no severity grading was collected. “Other” is an open residual option of the questionnaire, offered without a predefined list and without a free-text specification in the database, so its clinical content cannot be characterized. For the main analyses, disability was used dichotomously (any disability vs. none). For type-specific analyses, and following the reviewer-relevant consideration that people reporting more than one impairment may face greater limitations, we built six mutually exclusive categories: no disability; sensory only (visual or hearing, and no other type); physical only; cognitive only; multiple types (two or three of the sensory, physical or cognitive domains); and other only. This classification replaces the overlapping definition used previously, so that category percentages sum to 100%.

#### 2.3.2 Dependent variables (outcomes)

Two binary outcomes were analysed. (i) Physical inactivity: the survey general physical activity index, constructed by the Ministry of Sport, classifies respondents as active, partially active, or inactive from self-reported frequency, duration, and perceived intensity of physical activity across life domains (leisure, transport, occupational or domestic, and educational contexts), following the scoring protocol described in the survey documentation (14). We used the index as provided in the database and verified that its weighted marginal distribution reproduces the official figures of the executive report (14). Physical inactivity was defined as being classified as inactive (vs. active or partially active). (ii) Non-use of available spaces: among respondents reporting available spaces near the home, use for physical activity or sport was assessed dichotomously (yes/no); the outcome was not using them. Two further indicators were analysed descriptively as intermediate steps of the access chain: perceived availability of spaces (yes/no) and perceived distance to the nearest space (more than 30 min on foot vs. 30 min or less).

#### 2.3.3 Barriers

Non-users reported barriers from a closed multi-response list of eleven options (crime, entrance costs, drug use in the area, poor maintenance, overcrowding, inconvenient schedules, distance or difficult access, perceived or actual insecurity, inadequate lighting, lack of companionship, other), each analysed as a separate binary indicator. Perceived availability was assessed by asking whether sports facilities or adequate spaces for physical activity existed near the home; the term adequate and its examples (plazas, parks, cycle paths, multi-sport courts) are the literal wording of the questionnaire, and the item captures the reported existence of such spaces near the home rather than their effective availability for use. Response options ranged from less than 15 min on foot to more than 60 min, or no spaces. Fifty respondents with available spaces did not answer the use item and were treated as item non-response (Figure 1). For interpretation and international comparability, the eleven items were grouped a posteriori into four environmental dimensions consistent with the International Classification of Functioning, Disability and Health (17): physical and built environment (distance or difficult access, poor maintenance, inadequate lighting, overcrowding, inconvenient schedules, entrance costs), safety environment (crime, drug use, insecurity), social environment (lack of companionship), and unspecified (other).

#### 2.3.4 Covariates

Sex (female, male), age group (18-29, 30-39, 40-49, 50-59, 60-69, 70 years or over) and socioeconomic status (high, medium, low). Socioeconomic status is not derived from reported income: the survey documentation states that no official individual classification exists in Chile, and therefore assigns each respondent the level of their commune and zone of residence, complemented by questionnaire items on occupation and educational level; the three levels also served as sampling strata, with fixed quotas per region. Cut-off points and the exact allocation algorithm are not published by the survey, which we acknowledge as a limitation. Covariates were selected *a priori* rather than by statistical criteria: sex, age and socioeconomic status are plausible common causes of both disability and use of physical activity spaces, and were therefore treated as confounders and included in the primary models. Perceived distance was treated as a potential mediator rather than a confounder, because reported walking time may itself reflect mobility limitations, and region was treated as a contextual variable partly on the causal path; both were therefore examined in additional models only, and not in the primary adjustment set (Supplementary Figure 1).

### 2.4 Statistical analyses

Weighted prevalences were estimated with the normalized survey weight. Because the public database provides the survey weight but not stratum or cluster identifiers, full design-based variance estimation was not possible. Confidence intervals (95% CI) for prevalences were therefore computed with the Wilson method on an effective sample size derived from a Kish design effect of 2.51, calculated from the distribution of the survey weights as *n* squared over the sum of squared weights (effective *n* = 3,442 of 8,653). This single design effect is an approximation and may differ across estimates and subgroups; conclusions were unchanged when recomputing the key contrasts under design effects of 2.0 and 3.0. Differences between adults with and without disabilities in availability, distance, use and each barrier were tested with weighted Poisson regression with robust (HC1) variance, reported as crude prevalence ratios with 95% CI and *p* values, so that every comparison presented in Tables 1, 2 is accompanied by a formal test rather than by inspection of overlapping intervals. Adjusted associations were estimated as prevalence ratios (PR) using Poisson regression with robust (HC1) variance and survey weights; weighted HC1 errors do not reproduce design-based variance for a stratified multistage sample and should be read as an approximation. The primary models adjusted for sex, age group, and socioeconomic status; the primary non-use model, restricted to respondents with available spaces, estimates the total association without adjusting for distance, because reported walking time may itself reflect mobility limitations. Type-specific models used the six mutually exclusive categories, with no disability as the reference. Following a reviewer suggestion, we additionally examined effect-measure modification: weighted prevalences and crude PR were estimated within strata of age group (18-39, 40-59, 60 years and over) and sex, and multiplicative interaction was assessed with likelihood ratio tests comparing adjusted models with and without disability-by-age and disability-by-sex product terms (Supplementary Table 1); cells combining age and sex were examined descriptively. Additional models adjusted for distance (more than 30 min on foot) and for region (16 categories), and a sensitivity analysis excluded the unspecified (other) disability category. Analyses were conducted in Python 3.12.

**Table 1:** Characteristics of the study sample by disability status, ENAFyD 2024 (weighted percentages).

Characteristic	With disability (*n* = 1,006)	Without disability (*n* = 7,647)	Total (*n* = 8,653)
Sex			
Female	55.8	50.5	51.0
Male	44.2	49.5	49.0
Age group (years)			
18–29	15.1	22.4	21.7
30–39	14.9	23.0	22.2
40–49	8.5	18.1	17.2
50–59	16.5	16.9	16.9
60–69	23.2	12.1	13.1
70 or more	21.8	7.6	8.9
Socioeconomic status			
High	19.7	20.7	20.6
Medium	41.6	48.2	47.6
Low	38.7	31.0	31.7
Disability type (mutually exclusive)			
Sensory only	57.4	-	5.3
Physical only	28.6	-	2.7
Multiple types	6.1	-	0.6
Other only	4.8	-	0.4
Cognitive only	3.2	-	0.3
No disability	-	100.0	90.7

Weighted estimates using the normalized survey weight. Disability types are mutually exclusive: “multiple types” denotes two or three of the sensory, physical or cognitive domains reported by the same respondent (unweighted *n* = 52). Percentages within the disability group are computed over adults reporting any disability; percentages in the total column are computed over the whole sample. Percentages may not sum to exactly 100.0 because of rounding.

**Table 2:** Perceived availability, use, and barriers to the use of physical activity spaces by disability status, ENAFyD 2024 (weighted percentages, 95% CI) and crude prevalence ratios.

Indicator	With disability	Without disability	Crude PR (95% CI); p
Spaces available near home	92.1 (89.0–94.4)	91.4 (90.4–92.3)	1.01 (0.99–1.03); 0.478
Nearest space >30 min on foot	11.1 (8.4–14.6)	8.1 (7.2–9.1)	1.38 (1.12–1.70); 0.002
Physical inactivity	40.5 (35.8–45.4)	27.0 (25.5–28.6)	1.50 (1.37–1.64); <0.001
Non-use of available spaces	70.1 (65.2–74.6)	57.3 (55.4–59.2)	1.22 (1.16–1.29); <0.001
Barriers among non-users	*n* = 634	*n* = 3,635	
Distance or difficult access	14.0 (10.3–18.8)	7.7 (6.4–9.2)	1.82 (1.44–2.31); <0.001
Lack of companionship	25.9 (20.9–31.6)	16.8 (15.0–18.8)	1.54 (1.31–1.80); <0.001
Presence of crime	26.4 (21.3–32.2)	21.4 (19.4–23.6)	1.23 (1.06–1.44); 0.008
Inadequate lighting	15.3 (11.4–20.3)	12.2 (10.6–14.0)	1.25 (1.00–1.56); 0.046
Entrance costs	6.2 (3.8–9.9)	4.4 (3.5–5.6)	1.41 (0.98–2.03); 0.063
Inconvenient schedules	6.9 (4.4–10.7)	5.5 (4.4–6.8)	1.26 (0.89–1.77); 0.188
Drug use in the area	16.4 (12.3–21.5)	14.4 (12.7–16.3)	1.14 (0.93–1.40); 0.214
Insecurity in the area	19.8 (15.4–25.2)	20.1 (18.1–22.2)	0.99 (0.82–1.19); 0.883
Other	32.6 (27.1–38.6)	32.8 (30.4–35.3)	0.99 (0.87–1.13); 0.933
Overcrowding	5.4 (3.2–8.9)	7.6 (6.3–9.1)	0.71 (0.49–1.03); 0.074
Poor maintenance	19.2 (14.8–24.5)	23.5 (21.4–25.8)	0.82 (0.68–0.99); 0.035

Availability, inactivity, use, and barriers estimated among the relevant denominators (all adults; adults with a valid activity index; adults with available spaces; non-users with available spaces, multi-response). Barrier items were multi-response, so percentages do not sum to 100. 95% CI by the Wilson method on the effective sample size (Kish design effect 2.51). Crude prevalence ratios (adults with disabilities vs. adults without disabilities) from weighted Poisson regression with robust variance; barriers are ordered by the magnitude of the ratio.

A generative artificial intelligence tool supported analysis code development and language editing under the full supervision and responsibility of the authors, who verified all data, analyses, and references.

## 3 Results

### 3.1 Sample characteristics

Among the 8,653 adults surveyed, 1,006 reported at least one disability, corresponding to a weighted prevalence of 9.3% (95% CI 8.4-10.3). Using mutually exclusive categories, sensory disability alone was the most common (unweighted *n* = 577; 57.4% of the group with disabilities), followed by physical only (*n* = 279; 28.6%), multiple types (*n* = 52; 6.1%), other only (*n* = 53; 4.8%) and cognitive only (*n* = 45; 3.2%). Adults with disabilities were older than those without (45.0% vs. 19.7% aged 60 years or older), more frequently women (55.8% vs. 50.5%), and more frequently of low socioeconomic status (38.7% vs. 31.0%). The flow of participants through each analysis, including exclusions and item non-response, is presented in Figure 1: analyses of inactivity included 8,577 adults (76 with a missing activity index) and analyses of use included 7,659 adults with available spaces and a valid use response; covariates had no missing values. Sample characteristics by disability status are presented in Table 1.

### 3.2 Perceived availability of physical activity spaces

Perceived availability of sports facilities or adequate spaces near the home was virtually identical between groups: 92.1% (95% CI 89.0-94.4) of adults with disabilities and 91.4% (90.4-92.3) of adults without disabilities reported that such spaces existed, a difference that was not statistically significant (crude PR 1.01; 95% CI 0.99-1.03; *p* = 0.478). However, adults with disabilities more frequently reported that the nearest space was located more than 30 min away on foot (11.1%, 95% CI 8.4-14.6 vs. 8.1%, 7.2-9.1; crude PR 1.38; 1.12-1.70; *p* = 0.002) and, conversely, less frequently reported a space within 15 min on foot (55.7% vs. 58.4%).

### 3.3 Use of available spaces

Among adults who reported having spaces available, only 29.9% (95% CI 25.4-34.8) of those with disabilities used them for physical activity or sport, compared with 42.7% (40.8-44.6) of those without disabilities (crude PR for non-use 1.22; 1.16-1.29; *p* < 0.001) (Table 2). Use varied across mutually exclusive disability types; adjusted estimates for each type are presented in Table 3 and described in Section 3.5.

**Table 3:** Association between disability and physical inactivity and non-use of available spaces, ENAFyD 2024. Prevalence ratios (95% CI) from weighted Poisson regression with robust variance.

Model	Inactivity	Non-use of spaces
Any disability (primary model)	1.22 (1.11–1.33)	1.14 (1.08–1.20)
By mutually exclusive type		
Sensory only	1.17 (1.03–1.32)	1.09 (1.02–1.17)
Physical only	1.26 (1.09–1.46)	1.14 (1.05–1.24)
Cognitive only	1.13 (0.65–1.96)	1.29 (1.00–1.67)
Multiple types	1.24 (0.90–1.71)	1.47 (1.35–1.60)
Other only	1.48 (1.10–2.00)	1.20 (1.01–1.43)
Additional model: + distance (>30 min)	-	1.13 (1.07–1.19)
Additional model: + region (16)	1.13 (1.03–1.23)	1.15 (1.09–1.21)
Sensitivity: excluding other disability	1.20 (1.09–1.32)	1.13 (1.07–1.19)

Primary models adjusted for sex, age group, and socioeconomic status; the non-use model is restricted to adults with available spaces and estimates the total association without distance adjustment. Type-specific categories are mutually exclusive, with adults without disabilities as the common reference group; PR above 1 indicates higher prevalence among adults with the corresponding disability profile.

### 3.4 Barriers to the use of available spaces

Among non-users with available spaces (*n* = 634 with disabilities; *n* = 3,635 without), three barriers were significantly more frequent among adults with disabilities: distance or difficulty of access (14.0% vs. 7.7%; crude PR 1.82; 95% CI 1.44-2.31; *p* < 0.001), lack of companionship (25.9% vs. 16.8%; PR 1.54; 1.31-1.80; *p* < 0.001) and presence of crime (26.4% vs. 21.4%; PR 1.23; 1.06-1.44; *p* = 0.008); inadequate lighting was borderline (15.3% vs. 12.2%; PR 1.25; 1.00-1.56; *p* = 0.046). In contrast, lack of maintenance or deficient infrastructure was reported less frequently by adults with disabilities (19.2% vs. 23.5%; PR 0.82; 0.68-0.99; *p* = 0.035), and the remaining barriers, including the residual “other” category (32.6% vs. 32.8%; PR 0.99; 0.87-1.13; *p* = 0.933), did not differ significantly between groups. Table 2 presents the full barrier profile.

### 3.5 Association between disability and physical inactivity and non-use

The weighted prevalence of physical inactivity was 40.5% (95% CI 35.8-45.4) among adults with disabilities and 27.0% (25.5-28.6) among adults without disabilities (crude PR 1.50; 1.37-1.64; *p* < 0.001). In weighted Poisson models adjusted for sex, age group, and socioeconomic status, disability was associated with a 22% higher prevalence of physical inactivity (PR 1.22; 95% CI 1.11-1.33) and with a 14% higher prevalence of non-use of available spaces (PR 1.14; 1.08-1.20) (Table 3). Additional adjustment for distance yielded a similar estimate for non-use (PR 1.13; 1.07-1.19). In models using mutually exclusive categories, non-use was highest among adults reporting multiple disability types (PR 1.47; 1.35-1.60; *p* < 0.001), followed by cognitive only (1.29; 1.00-1.67; *p* = 0.049), other only (1.20; 1.01-1.43), physical only (1.14; 1.05-1.24) and sensory only (1.09; 1.02-1.17). For inactivity, associations were significant for other only (1.48; 1.10-2.00), physical only (1.26; 1.09-1.46) and sensory only (1.17; 1.03-1.32), whereas estimates for multiple types (1.24; 0.90-1.71) and cognitive only (1.13; 0.65-1.96) were imprecise and compatible with no association, reflecting the small size of these groups. Excluding unspecified disabilities gave similar results (inactivity PR 1.20; 1.09-1.32; non-use PR 1.13; 1.07-1.19). Additional adjustment for region attenuated the association with inactivity (PR 1.13; 1.03-1.23) and left non-use essentially unchanged (PR 1.15; 1.09-1.21).

### 3.6 Stratified analyses by age and sex

The similarity of perceived availability held in every stratum examined (crude PR between 0.99 and 1.03, all *p* > 0.10; Supplementary Table 1). The disability gap in non-use of available spaces was consistent across age groups (interaction *p* = 0.551) and sexes (interaction *p* = 0.848), being clearest at ages 40-59 (74.3% vs. 58.4%; PR 1.27; 1.16-1.39). For physical inactivity, in contrast, the gap differed by sex (interaction *p* = 0.009): the disparity was larger among men (40.2% vs. 22.6%; crude PR 1.78; 1.55-2.05) than among women (40.8% vs. 31.4%; PR 1.30; 1.15-1.47), because inactivity among adults with disabilities converged near 40% in both sexes while men without disabilities were substantially more active than women without disabilities. Barrier profiles also varied: lack of companionship was differential mainly among adults aged 60 and over (30.5% vs. 19.4%; PR 1.58; 1.26-1.98) and among men (27.7% vs. 14.2%; PR 1.96; 1.54-2.49), whereas the presence of crime was differential only among women (30.9% vs. 24.3%; PR 1.27; 1.06-1.53) and at ages 40-59. Distance or difficult access was elevated in nearly every stratum. Cells combining age and sex are small for the group with disabilities (unweighted *n* = 73 to 232) and are reported descriptively in Supplementary Table 1; among them, women aged 18-39 with disabilities showed a marked gap in non-use (71.9% vs. 55.8%) whereas men of the same age did not (43.7% vs. 47.9%).

## 4 Discussion

### 4.1 Main findings

In this nationally representative sample of Chilean adults, disability was not associated with lower perceived availability of physical activity spaces: more than nine out of ten adults in both groups reported having plazas, parks, cycle paths, or sports facilities near their homes, with no statistically significant difference between them (92.1% vs. 91.4%; crude PR 1.01; 95% CI 0.99-1.03; *p* = 0.478). The disparity was concentrated in the use of those spaces: only 29.9% of adults with disabilities used available spaces, compared with 42.7% of their counterparts without disabilities (crude PR for non-use 1.22; 1.16-1.29; *p* < 0.001), with heterogeneity across mutually exclusive disability types and the largest adjusted estimate among adults reporting multiple types. After adjustment for sex, age, and socioeconomic status, disability remained associated with both physical inactivity (PR 1.22; 95% CI 1.11-1.33) and non-use of available spaces (PR 1.14; 1.08-1.20). Taken together, these findings indicate that in Chile the disability gap is concentrated in the use of existing spaces rather than in their perceived availability; whether this reflects functional inaccessibility, which was not directly measured, is a hypothesis these data raise but cannot confirm. An important nuance is that the availability item captures the perceived existence of spaces near the home, not their effective availability for use: within these same data, adults with disabilities more often reported that existing spaces were farther away and that access was difficult, so similar perceived existence should not be read as equal opportunity to use those spaces.

### 4.2 Similar perceived availability, lower use

The central finding of this study is the dissociation between perceived availability and actual use. The magnitude of the inactivity gap is consistent with the international pattern: in the United States, 47.1% of adults with disabilities aged 18 to 64 were physically inactive compared with 26.1% of those without disabilities in 2009-2012 (18), and more recent US estimates confirm persistently lower sufficient activity in subpopulations with disability (19), figures remarkably close to the 40.5% (95% CI 35.8-45.4) and 27.0% (25.5-28.6) observed here. In Australia, among 54,343 adults surveyed, the 15% who reported a disability participated less in leisure-time physical activity across the entire lifespan (20), and in a Dutch registry-linked sample of 321,656 adults every disability type examined was associated with lower activity levels (21). Within the region, a population-based study in the city of Sao Paulo found 22.4% of adults without disabilities active in leisure time against 10.9% of those with physical disability, a 40% lower prevalence, with smaller differences for visual (17.8%) and hearing (20.3%) disability (22), a gradient strikingly similar to ours. In Chile, the pandemic-era disability survey had already documented that only 11.9% of adults with disabilities met guidelines (11); the present study extends that evidence by separating perceived availability from use. Nine out of ten Chilean adults with disabilities perceive that spaces exist, yet fewer than one in three uses them. This pattern echoes the distinction raised two decades ago by Rimmer and colleagues, whose focus groups across the United States identified 178 barriers showing that the mere existence of fitness and recreation facilities does not guarantee their usability for people with disabilities (8), a situation that facility audits show has persisted (7). The attenuation of the crude inactivity gap (from roughly 13.5 percentage points to PR 1.22 after adjustment) reflects the older age structure of the population with disabilities, but the persistence of the association after adjustment indicates a disability-specific disadvantage beyond demographic composition.

### 4.3 Barriers and heterogeneity across disability types

The barrier profile among non-users reinforces this interpretation. The three barriers significantly more frequent among adults with disabilities were distance or difficulty of access (PR 1.82), lack of companionship (PR 1.54) and presence of crime (PR 1.23), while complaints about maintenance were significantly less frequent in this group (PR 0.82). This asymmetry is not easily explained by a uniformly more negative perception of the environment, although differential perception or reporting cannot be ruled out: the excess concentrates in barriers plausibly related to functional limitations, namely reaching the space and having support once there. These comparisons are descriptive; barriers were assessed only among non-users and no mediation analysis linking them to the disability and non-use association was performed. Two items deserve particular caution in interpretation: distance or difficult access conflates geographic distance with inaccessibility of the space itself, and lack of companionship may reflect the need for assistance among people with higher support needs or social isolation among more independent adults, two different policy problems that a single item cannot distinguish. Each differential barrier has direct support in the literature. A meta-analysis estimated 27% greater odds of achieving high physical activity when people feel safe from crime and 28% lower odds in high-crime areas (23), and in Chile perceived recreational safety has been identified as a barrier in national data on play environments (24). Companionship support has emerged as the most influential form of social support for physical activity in a scoping review of 116 studies (25), which is directly relevant to a group in which 45.0% are aged 60 or over. These barriers may also weigh unevenly within the group: women represent 55.8% of adults with disabilities in our sample, and a systematic review focused specifically on women with physical disabilities identified eight clusters of gender-specific barriers, concluding that participation depends on an inclusive social environment rather than individual disposition alone (26). The heterogeneity across mutually exclusive disability types is informative in the same direction. Adults reporting multiple types showed the strongest association with non-use (PR 1.47), consistent with the expectation that coexisting impairments compound environmental demands, followed by cognitive only (1.29), physical only (1.14) and sensory only (1.09). This ordering mirrors the type-specific barrier structure reported elsewhere: among Spanish users of adapted sport programs, environmental barriers predominated in visual disability while intrinsic barriers predominated in physical and intellectual disability (27); environmental obstacles and inaccessible infrastructure are consistent correlates of low activity in sight loss (28); and critically low activity levels have been documented in intellectual disability (29). The overall pattern is consistent with the global estimate that people with disabilities are 16% to 62% less likely to meet physical activity guidelines, a range that reflects precisely this heterogeneity across disability types and contexts (3).

The stratified analyses add a population-health reading to this heterogeneity. The only significant effect-measure modification involved sex and inactivity: disability appears to erase the male advantage in physical activity observed in the general population, so that men and women with disabilities converge at similarly high inactivity (around 40%) despite very different baselines. The barrier profiles are consistent with distinct mechanisms across groups: companionship weighs most among older adults and among men, safety concerns weigh among women, and distance cuts across strata. Age patterns should be read with caution: because the survey is cross-sectional, differences across age groups blend life-course processes (disability acquired with ageing) with generational differences in activity habits and cannot be interpreted as the causal effect of ageing.

### 4.4 Implications for policy and practice

Investment in new infrastructure, the dominant policy response to physical inactivity, would not by itself address the disparity documented here, since perceived availability is already near universal in both groups. The actionable margins are different: accessibility audits and universal design retrofitting of existing spaces, safe route programs connecting homes to spaces, and accompanied or peer-supported physical activity schemes, which directly target the three differential barriers identified. These directions align with the World Health Organization guidelines for people living with disability and the Global Action Plan on Physical Activity, which call for inclusive environments and the removal of access barriers rather than generic promotion alone (2, 4, 30). These directions find a concrete policy window in Chile: the newly enacted National Policy on Physical Activity and Sport 2026-2037, which itself acknowledges, citing the national disability survey, that 79.3% of persons with disabilities engage in no physical activity, delegates its quantified targets to forthcoming national and regional plans, and the present findings suggest which levers deserve attention in those plans, pending studies that measure accessibility directly (10).

A further implication concerns human resources. Making existing spaces usable requires professionals prepared to receive people with disabilities: training physical education teachers, sport coaches, and municipal staff in inclusive and adapted physical activity is a comparatively low-cost complement to infrastructure investment, and one that the implementation plans of the 2026-2037 national policy could incorporate explicitly (10).

### 4.5 Strengths and limitations

Strengths of this study include the national probabilistic sample, the weighted design, and the joint measurement of availability, use, and barriers within the same instrument, which allows the disparity to be located along the access chain. Several limitations should be considered. First, disability was self-reported through a brief dichotomous item battery without severity grading or use of the standardized Washington Group questions (31). This has two implications for the direction of bias: the battery likely captures the more severe or more self-identified cases, which would tend to accentuate the contrasts observed for sensory and physical disability, while milder unreported limitations remain in the comparison group, diluting the population-level difference; and the cognitive only group was small (*n* = 45), yielding wide confidence intervals that reflect limited statistical power, warrant cautious interpretation, and preclude firm type-specific conclusions. The residual “other” category could not be characterized, since the questionnaire offers it without a predefined list or free-text specification, so the conditions grouped there remain unknown; results excluding this category were nevertheless unchanged. In addition, no documented adaptation of the questionnaire for respondents with cognitive disability was available and comprehension checks were not reported, so differential measurement error in this group cannot be excluded, and its composition is likely heterogeneous in severity. Second, the public database provides the survey weight but not stratum or cluster identifiers; variance was approximated with a single Kish design effect of 2.51 derived from the weights, an approximation whose adequacy may vary across estimates, although conclusions held under design effects between 2.0 and 3.0. Socioeconomic status was assigned from the commune and zone of residence rather than from individual income, and its cut-off points are not published by the survey, so residual confounding by individual socioeconomic position cannot be ruled out. Accessibility itself (adapted equipment, accessible entrances and toilets, inclusive programming, support staff) was not measured, which is the most direct explanation the survey cannot test and a plausible component of the large unspecified (other) barrier category (32.6%). Third, the cross-sectional design precludes causal inference: lower use may also shape perceived barriers rather than only the reverse. Fourth, 32.6% of non-users with disabilities selected other as a barrier, indicating that the closed list did not capture part of the relevant barrier space, possibly including disability-specific obstacles such as inaccessible equipment or lack of adapted programming. Fifth, all measures were self-reported. The magnitude of our prevalence estimate supports the reading that the battery captures mainly more severe cases: the III National Disability Study (ENDISC 2022), which measures disability in adults with a graded capacity-performance approach based on the ICF, estimates that 17.6% of Chilean adults live with some degree of disability (6.2% mild to moderate and 11.4% severe), so the 9.3% captured by the ENAFyD dichotomous battery lies closer to the severe fraction than to the total (32). External consistency is nevertheless reassuring: adults aged 60 or over represent 43.4% of the population with disabilities in ENDISC and 45.0% in our sample, and women predominate in both sources. The survey also does not distinguish congenital, acquired, or ageing-related disability, so the observed age gradient blends life-course and generational processes; and because ENDISC does not classify by impairment type, our type-specific estimates have no official national benchmark, reinforcing the case for incorporating standardized measures such as the Washington Group questions in future ENAFyD waves (31, 32). Future studies should examine each disability type separately, with adequately powered samples and standardized severity measures.

## 5 Conclusion

In Chilean adults, disability is associated with higher physical inactivity and with substantially lower use of physical activity spaces despite similar perceived availability. Among non-users, barriers related to distance and access, companionship, and safety were more frequently reported by adults with disabilities. These findings indicate that the usability of existing spaces, and not only the construction of new ones, deserves priority attention in the implementation plans of the new national policy. In practical terms, the priority is not building more spaces but making the existing ones usable: accessible, reachable, safe, and socially supported. Confirming the mechanisms behind this gap requires studies that measure accessibility directly.

## Data Availability

The ENAFyD 2024 databases are available from the Ministry of Sport of Chile.

## Supplementary material

Supplementary Figure 1 Directed acyclic graph summarizing the assumed relationships among disability, covariates, perceived distance, region, and the study outcomes.

Supplementary Table 1 Perceived availability, physical inactivity, non-use of available spaces, and selected barriers by disability status, stratified by age group and sex, ENAFyD 2024. Weighted percentages and crude prevalence ratios (95% CI); p values.
